# MASLD-Related Hepatocarcinoma: Special Features and Challenges

**DOI:** 10.3390/jcm13164657

**Published:** 2024-08-08

**Authors:** Carmen Yagüe-Caballero, Diego Casas-Deza, Andrea Pascual-Oliver, Silvia Espina-Cadena, Jose M. Arbones-Mainar, Vanesa Bernal-Monterde

**Affiliations:** 1 Gastroenterology Department, Miguel Servet University Hospital, 50009 Zaragoza, Spain; carmenyaguecaballero@gmail.com (C.Y.-C.); diegocasas8@gmail.com (D.C.-D.); apascualoliver@gmail.com (A.P.-O.); silespina@gmail.com (S.E.-C.); vbernal@salud.aragon.es (V.B.-M.); 2 Adipocyte and Fat Biology Laboratory (AdipoFat), Translational Research Unit, Miguel Servet University Hospital, 50009 Zaragoza, Spain; 3 Instituto de Investigación Sanitaria (IIS) Aragon, 50009 Zaragoza, Spain; 4 Instituto Aragones de Ciencias de la Salud (IACS), 50009 Zaragoza, Spain; 5 CIBER Fisiopatología Obesidad y Nutrición (CIBERObn), Instituto Salud Carlos III, 28029 Madrid, Spain

**Keywords:** NAFLD, NASH, cirrhosis, hepatocellular carcinoma, surveillance, prevention

## Abstract

Metabolic-associated steatohepatitis liver disease (MASLD) currently impacts a quarter of the global population, and its incidence is expected to increase in the future. As a result, hepatocellular carcinoma associated with MASLD is also on the rise. Notably, this carcinoma does not always develop alongside liver cirrhosis, often leading to a more advanced stage at diagnosis. The challenge lies in accurately identifying patients who are at a higher risk to tailor screening processes effectively. Additionally, several therapeutic approaches are being explored to prevent hepatocellular carcinoma, although there are no universally accepted guidelines yet.

## 1. Metabolic Dysfunction-Associated Steatotic Liver Disease (MASLD)

### 1.1. Concept and Nomenclature

For decades, hepatic steatosis closely linked to obesity has been known to be a cause of liver damage, potentially leading to liver cirrhosis. In the 1980s, it was first recognized as an independent entity associated with liver damage and was named non-alcoholic steatohepatitis [1]. Non-alcoholic fatty liver disease (NAFLD) encompasses a spectrum of chronic liver pathologies, from isolated non-alcoholic fatty liver (NAFL) to non-alcoholic steatohepatitis (NASH), characterized by inflammation and tissue damage, with or without fibrosis [2].

Over the past few decades, research into this disease and its underlying mechanisms has led to its recognition as a multisystem disorder. Consequently, in 2020, its name was updated to metabolic-associated fatty liver disease (MAFLD). Diagnosing MAFLD requires histological proof, imaging tests, or biomarkers that demonstrate excessive fat accumulation in the liver, accompanied by at least one of the following: overweightness or obesity, type 2 diabetes mellitus, or metabolic dysfunction [3]. This shift in nomenclature transitioned MASLD from an exclusionary diagnosis to an inclusive one. In 2023, the term was revised once more to metabolic dysfunction associated with hepatic steatosis (MASLD) to better represent the pathophysiological roots of the condition and its strong association with cardiometabolic changes [4]. Furthermore, the latest consensus on liver disease nomenclature has introduced a new subgroup known as MetALD. This category includes patients with MASLD who consume alcohol at levels considered harmful, defined as 210 to 420 g per week for men and 140 to 350 g per week for women [5,6]. Recent studies with huge historic cohorts have compared MASLD with NAFLD in terms of disease prevalence and outcomes. It seems that both definitions show a great concordance and include basically the same population. Therefore, previous data generated for NAFLD could be used indistinctly for MASLD.

### 1.2. Epidemiology

The increasing incidence of metabolic-associated fatty liver disease (MASLD) mirrors the worldwide surge in obesity, which has nearly tripled over recent decades [7]. A meta-analysis conducted by Younossi et al. indicates that MASLD affects approximately 30% of the global population [8]. This study identifies Latin America as having the highest prevalence at 44% (CI: 30.66–59%), followed by western Europe, with a prevalence estimated at 25.1% (20.55–30.28%) [8]. Moreover, available evidence suggests that nearly all patients diagnosed with non-alcoholic fatty liver disease (NAFLD), ranging between 98 and 99%, also meet the criteria for MASLD [5,6].

### 1.3. Etiopathogenesis

**Figure 1:**
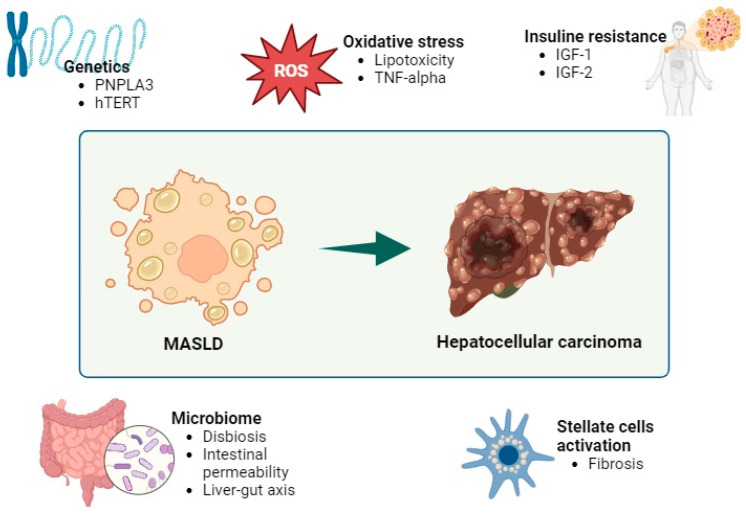
Etiopathogenic pathways from hepatocellular carcinoma (HCC) to MASLD.

MASLD is closely associated with metabolic syndrome, as indicated in several studies [9]. Despite ongoing research, the precise etiopathogenic mechanisms of MASLD remain incompletely understood. Historically, the “two-hit” hypothesis was the predominant theory explaining the etiopathogenesis and progression of MASLD [10]. However, this theory has been superseded by the “multiple hits” hypothesis, which offers a more comprehensive explanation [11]. According to the “multiple hits” hypothesis, MASLD develops in genetically predisposed individuals through the interaction of various environmental factors, including lifestyle choices, dietary habits, and microbiota composition (Figure 1). Key metabolic alterations, particularly in carbohydrate and lipid metabolisms and insulin resistance, play critical roles. These metabolic changes include increased de novo hepatic lipogenesis, impaired inhibition of adipose tissue lipolysis, enhanced release of fatty acids, and reduced fatty acid oxidation, leading to lipotoxicity [11]. Lipotoxicity triggers oxidative stress, which, in turn, causes mitochondrial dysfunction and initiates inflammatory cascades marked by the increased production of cytokines and other pro-inflammatory mediators, such as NLRP3 [12,13]. The resulting oxidative stress increases hepatocyte susceptibility to lipotoxic death, releasing inflammatory mediators and activating multiple inflammatory pathways, including those involving tumor necrosis factor (TNF), a key molecule in the progression to fibrosis [14]. This process is exacerbated by insulin resistance [11]. Chronic inflammation within the liver causes cellular damage and fibrosis, affecting the function of Kupffer cells and stellate cells. Furthermore, the inflammation extends beyond the liver, leading to chronic low-grade systemic inflammation, which increases the risk of long-term complications, such as cardiovascular events and the development of neoplasias [14,15].

## 2. MASLD-Related Complications

### 2.1. Cardiovascular Complications

Cardiovascular complications are a major concern for MASLD patients and represent the leading cause of mortality, particularly in those who have not progressed to cirrhosis [15]. The connection between MASLD and cardiovascular disease is substantial and well-documented. A national cohort study in Japan involving approximately 4 million individuals showed that MASLD patients experience more than twice the incidence of cardiovascular events, compared to healthy individuals [16]. MASLD is closely associated with several key cardiovascular risk factors, such as type 2 diabetes, obesity, and dyslipidemia [15].

Several physiological mechanisms underpin the heightened cardiovascular risk in MASLD patients. These include chronic low-grade inflammation, which contributes to vascular damage and atherosclerosis; increased oxidative stress, which exacerbates endothelial dysfunction and promotes arterial plaque formation; hepatic insulin resistance, which disrupts glucose and lipid metabolisms, further exacerbating cardiovascular risk; and dysregulated lipid metabolism, characterized by high low-density lipoprotein (LDL) and low high-density lipoprotein (HDL) levels, which are directly involved in atherogenesis [15,17].

Moreover, MASLD is part of the broader metabolic syndrome, often presenting multiple cardiovascular risk factors concurrently. This syndrome is considered an independent risk factor for cardiovascular disease, which is evidenced by typical dyslipidemia profiles in MASLD patients that increase atherogenic potential [15]. Furthermore, hepatic steatosis and fibrosis in MASLD patients are linked to diastolic dysfunction and contribute to coronary artery plaque development [16]. The pathogenic bridge to type 2 diabetes mellitus in MASLD involves the accumulation of lipids, such as ceramides and diacylglycerols, which are known to induce insulin resistance in liver tissues through specific mediators. These lipids accumulate due to increased dietary intake, insulin resistance in skeletal muscle common in sedentary lifestyles, and mitochondrial dysfunction [18].

Despite the established link between MASLD and cardiovascular risks, standardized screening and primary prevention protocols remain underdeveloped, highlighting the need for continued research in this area.

### 2.2. Extrahepatic Neoplasms

The most frequent extrahepatic neoplasms in patients with MASLD are endometrial, breast, prostate, colorectal, and lung cancers [19]. To date, it has not been demonstrated that the risk increases in the presence of a higher degree of fibrosis or cirrhosis [19]. As the incidence of MASLD is expected to increase in the coming decades, the incidence of its associated neoplasms is also expected to rise [8].

The most studied extrahepatic neoplasm associated with MASLD in literature to date is colorectal cancer. In the pathogenesis of this cancer associated with MASLD, insulin resistance and chronic low-grade inflammation are thought to play crucial roles, with a proliferative effect and the inhibition of apoptosis, thus initiating the process of carcinogenesis [19,20]. Numerous studies have shown the increased risk of adenomas and colorectal cancer in patients with MASLD. MASLD has been recognized as an independent risk factor for the development of colon polyps. It has been shown that in this population, the risk of developing adenomatous colon polyps is three times that of the general population. Moreover, there are more likely to be multiple polyps, and they are more likely to be located in the right or transverse colon and present high-grade dysplasia [19]. In a study conducted in the USA in a cohort of 19.163 subjects, MASLD was associated with an increased risk of colon cancer (IRR 1.8; 95% CI, 1.1–2.8). In a prospective study by Cho et al., MASLD was independently associated with an increased risk of developing adenomatous polyps (OR 2.76; 95% CI, 1.51–5.06; *p* = 0.001) [20]. Hence, it is suggested that a more rigorous colorectal cancer screening protocol be implemented for MASLD patients, given that the right colon is the most common site for neoplasia in this group.

Establishing the link between MASLD and other malignancies is more complex. MASLD may impact their development through low-grade chronic inflammation and insulin resistance. Hyperinsulinemia increases the levels of IGF-1, which has proliferative and anti-apoptotic effects. High levels of IGF-1 have been associated with prostate, colorectal, lung, and breast cancers. It is also believed that dysbiosis plays a role in the development of several neoplasms [19]. In a metanalysis that included 10 cohort studies (182.202 patients and 8.500 cases of extrahepatic cancers), MASLD was associated with a 1.5–2-fold increased risk of gastrointestinal cancers (including colorectal cancer) and a 2.5-fold increased risk of thyroid cancer. It was also related to a significant increase in the risk of breast, lung, urinary, or gynecological cancer (between 1.2 and 1.5) [8]. Moreover, obesity is a key factor in the onset of esophageal, pancreatic, breast, and thyroid cancers [19], potentially serving as the link between MASLD and these neoplasms.

Given these findings, it is crucial to conduct further studies to identify MASLD patients at high risk for extrahepatic neoplasms and to develop effective screening strategies. This effort will help in the early detection and management of cancer in this vulnerable population [19,20].

## 3. Hepatocellular Carcinoma and MASLD

### 3.1. Pathogenesis

As of now, the precise etiopathogenic pathways linking hepatocellular carcinoma (HCC) to MASLD remain elusive. Insulin resistance stands out as a pivotal factor in carcinogenesis. Insulin resistance and subsequent hyperinsulinemia contribute to elevated levels of insulin-like growth factor 1 (IGF-1), which, in turns, promotes cell survival, stimulates cell proliferation, and facilitates angiogenesis. Additionally, albeit to a lesser degree, IGF-2, which shares similar biological effects with IGF-1, is also elevated [21].

There is also an increase in oxidative stress, partly favored by hyperinsulinemia. Insulin promotes the deposition of lipids in the liver and increases the oxidation of free fatty acids, producing an increase in reactive oxygen species (ROS) [11]. The increase in ROS produces the peroxidation of the lipid membrane with mitochondrial, and therefore cellular, damage [22]. This results in inflammation, necrosis, and activation of liver stellate cells that favor fibrogenesis and have a carcinogenic role [21,23]. In addition, the increase in ROS leads to an increase in the production of pro-inflammatory cytokines, including tumor necrosis factor alpha, which activates pro-oncogenic pathways. The proinflammatory environment produces an increase in hepcidin, which decreases iron absorption and iron efflux from hepatocytes and macrophages. The increases in iron inside hepatocytes favors inflammation and carcinogenesis [23].

The intestinal microbiota plays a fundamental role in the development of MASLD and its progression to HCC. In patients with MAFLD, there is an increase in intestinal permeability, due to the decrease in intercellular junctions between enterocytes [11,24]. This increase in permeability results in bacterial translocation and other products that promote inflammation [11,24]. The microbiota interacts with the immune system through the liver–intestine axis. Patients with MASLD experience a reduction in microbial species diversity within the gut microbiota [23]. Moreover, those with HCC related to MASLD exhibit an elevation in pathogenic genera, primarily Bacteroides and Ruminococcaceae. A reduction in protective genera, such as Akkermansia and Bifidobacterium, has also been seen, which, in murine models, seem to favor the integrity of the intestinal barrier and reduce hepatic inflammation [24]. In addition to contributing to perpetuating chronic inflammation, the greater conversion of primary to secondary bile acids affects the antitumor response of natural killer lymphocytes and produces an increase in fibroblast growth factor (FGF)-19, which is involved in the proliferation of hepatocytes [11,23,24,25].

Research into genetic factors contributing to the pathogenesis of HCC associated with MASLD is ongoing. Currently, the genetic variant most strongly linked to an increased risk of HCC progression is the single nucleotide polymorphism rs738409 of the “patatin-like phospholipase-3” (*PNPLA3*) gene, resulting in the I148M isoform of its encoded protein [11]. The frequency of this variant ranges from 17 to 49% across different ethnicities and geographic regions, with a higher prevalence observed in Hispanic populations [26]. The protein adiponutrin, encoded by the *PNPLA3* gene, plays a role in lipid export from the liver, and the nucleotide substitution (isoleucine to methionine) disrupts protein function, leading to enhanced fat accumulations in hepatocytes and stellate liver cells, thereby promoting carcinogenesis [11,21]. Carriers of this variant face an elevated risk of developing cirrhosis and hepatocellular carcinoma, with homozygotes facing an even higher risk [11,21,26]. Additionally, ongoing investigations involve other genes, such as human telomerase reverse transcriptase (*hTERT*). The shortening of telomeres in peripheral blood and the presence of *hTERT* mutations correlate with the risk of HCC development in MASLD subjects, though further research is warranted in this area [21]. In addition, recent studies demonstrated the involvement of epigenetic mechanisms in the regulation of Tubb2b expression in the pathogenesis of NASH-HCC [21,22].

### 3.2. Epidemiology

Primary liver neoplasms are the sixth most common type of cancer and rank third among the most frequent causes of cancer-related mortality [21]. HCC is the most common primary liver neoplasm, representing between 75 and 85% of all cases [27]. The highest incidences are recorded in Asia and Africa, with Mongolia having the highest incidence rate worldwide [27]. In these countries, HCC is associated with the hepatitis B virus.

Among MASLD patients, an estimated 20–30% progress to steatohepatitis associated with metabolic dysfunction (MASH), with 10–20% of MASH patients advancing to liver cirrhosis [27]. In MASLD-induced cirrhosis cases, the incidence of HCC ranges from approximately 0.5% to 2.6% [21,24]. With MASLD affecting more than a quarter of the global population and with its prevalence rapidly rising alongside obesity rates, MASLD is poised to become the leading cause of liver disease. Consequently, an increase in MASLD-related HCC cases is anticipated [21,24]. Conversely, other etiologies of HCC, such as hepatitis B and C viruses, are declining due to vaccination and treatment strategies [21,22,28].

The primary risk factor for HCC development in MASLD patients is the presence of liver cirrhosis, though it can occur even without cirrhosis [21]. Risk is higher in patients with decompensated cirrhosis, compared to those without prior decompensations [29]. Additionally, the emergence of HCC is closely linked to metabolic syndrome, with obesity—particularly central obesity—identified as an independent risk factor [21]. Type 2 diabetes mellitus exacerbates liver disease progression and HCC development by perpetuating chronic inflammation and oxidative stress. Notably, diabetes poses a higher HCC risk in men than in women and elevates the risk with a longer disease duration [21,27,30].

In a prospective study by Chen et al., the risk of HCC development was compared among patients with steatosis due to MASLD, MetALD, and alcohol-induced liver disease versus those with cirrhosis but without steatosis or other cardiovascular risk factors. The findings revealed a higher HCC risk in patients with steatosis, particularly in the MetALD subgroup (hazard ratio: 2.91, CI: 2.11–4.03), compared to non-SLD without cardiometabolic risk factors [31].

### 3.3. Clinical Presentation

In various studies investigating HCC related to MASLD, age, sex, and comorbidities emerge as significant factors. Typically, MASLD-related HCC is diagnosed at an older age, with an average age of 73 years, compared to 66 and 70 years for hepatitis C and B, respectively [24]. In a meta-analysis conducted by Hao Tan et al. encompassing 61 studies and a total of 94,636 HCC patients (15,377 with MASLD-related HCC and 79,259 with HCC from other causes), no significant gender differences were noted [28]. However, other research indicates a higher prevalence in men [27]. MASLD-related HCC patients often exhibit a higher burden of comorbidities, notably hypertension, diabetes, and dyslipidemia, and are more likely to present with cardiovascular pathology at the time of diagnosis [11,28]. Additionally, they tend to have a higher body mass index, frequently demonstrating overweightness or obesity [28,32].

Moreover, HCC related to MASLD tends to manifest with larger lesions, with an average difference of 0.67 cm (95% CI 0.35–0.98, *p* = 0.0087). Additionally, lesions are more commonly uninodular [28]. Lastly, in an observational study in Italy involving 6882 HCC patients diagnosed between 2002 and 2019, MASLD emerged as the leading cause of HCC. Despite being diagnosed at a more advanced stage compared to other causes, MASLD-related HCC exhibited lower mortality, suggesting a comparatively less aggressive disease course [33].

### 3.4. Screening

A notable feature of HCC related to MASLD is that approximately one-third of cases occur in patients without liver cirrhosis, setting it apart from HCC related to other liver diseases, like viral hepatitis or autoimmune conditions [24,27].

In a meta-analysis comprising 19 studies and 168,571 patients, the prevalence of HCC related to MASLD without cirrhosis was 38%, whereas the prevalence of HCC without cirrhosis from other etiologies (e.g., alcohol or viral hepatitis) was 14.2% [24,34]. One plausible explanation for this clinical presentation is that in a non-cirrhotic liver, there is less resistance to tumor expansion. Moreover, individuals with MASLD but without liver cirrhosis are not typically included in HCC screening programs, potentially leading to delayed lesion detection [28].

The aim of screening for HCC is twofold: to reduce mortality attributed to HCC and to facilitate early detection of the neoplasia. To achieve these goals, accurate identification of the at-risk population is crucial. Presently, guidelines recommend HCC screening via abdominal ultrasound every 4 to 6 months for all patients with liver cirrhosis, regardless of etiology [35,36]. Abdominal ultrasound offers the advantages of being non-invasive and radiation-free. However, its efficacy is contingent upon the operator’s skill, and in individuals with significant central obesity, its sensitivity may be compromised. For patients with challenging ultrasound evaluations, alternative imaging techniques may be considered.

A particularity of HCC related to MASLD that differentiates it from other etiologies is that up to 40% of the cases develop in the absence of liver cirrhosis. The occurrence of HCC in MASLD patients without cirrhosis is relatively rare. Given the rapid increase in MASLD cases, screening all MASLD patients would be financially prohibitive. Therefore, at present, there is no justification for universal screening in all cases.

Today’s challenge lies in identifying, within the subset of patients lacking liver cirrhosis, those at high risk and optimizing screening systems [37]. While obesity and type 2 diabetes are established risk factors for HCC development, their roles in non-cirrhotic patients remain unclear. Liver fibrosis severity correlates with increased risks of HCC and mortality, prompting the development of tools to detect advanced fibrosis [29,37]. However, the nonlinear evolution of liver fibrosis complicates assessment. Liver biopsy, the gold standard for fibrosis determination, is invasive and prone to adverse effects and inter-observer variability, making non-invasive markers preferable for screening [29,37].

Several non-invasive tests utilizing blood or serum markers or ultrasound techniques are currently accessible and are progressively employed for assessing liver fibrosis. Among those based on blood markers, alpha-fetoprotein (AFP) is widely studied as an HCC tumor marker, but its efficacy for early detection is debated, due to varying sensitivities and potential false positives. While European guidelines recommend biannual ultrasound screening without AFP, American guidelines advocate combined screening to enhance ultrasound efficiency [35,36]. The Fibrosis-4 index (FIB-4) offers a simple, cost-effective tool for identifying high-risk HCC patients, showing a good predictive ability for HCC in MASLD populations [35,36,37]. A study conducted in a large European cohort showed that the risk of developing HCC was higher as the FIB-4 score increased. Patients with FIB-4 scores of 1.30–2.67 and >2.67 had a risk ratio of 3.74 and 25.2, respectively, compared to patients with a FIB-4 < 1.3 [37]. The non-alcoholic fatty liver disease (NAFLD) fibrosis score demonstrates superior predictive capability for HCC, compared to other fibrosis indices [29,37,38,39,40]. The GALAD score, also based on blood markers along with age and sex, exhibits a promising diagnostic capacity for HCC, providing specificity compared to AFP. In a case-control study carried out in patients with MASLD, the GALAD score had a sensitivity of 68% and a specificity of 95% for the early diagnosis of HCC. In the prospective arm, this score identified patients who will develop HCC a year and a half before their diagnosis [41]. The NAFLD fibrosis score (NFS) is an extensively used non-invasive test for staging liver fibrosis in patients with MASLD. It has also been proposed as a screening test for MASLD patients at risk of HCC. In a study that compared several non-invasive tests in 1173 patients with MASLD, NFS showed a good ability to predict the development of HCC (AUROC = 0.889 ± 0.048) [37].

Hepatic elastography, such as controlled transient elastography (VCTE), offers a valuable fibrosis assessment, but interpretation can be challenging within indeterminate risk zones [37,40,41,42,43]. Unfortunately, both serological markers and elastographic tests have a gray area of indeterminate risk that further complicates decision-making [29]. Recently, the liquid biopsy has emerged as a potential tool for early HCC detection, with circulating tumor DNA, cells, and extracellular vesicles showing promise, compared to serum AFP levels [44]. On the other hand, genetic studies for high-risk patient detection currently lack sensitivity, specificity, and cost-effectiveness [44].

The latest guideline from the European Association for the Study of the Liver (EASL) suggests evaluating inclusion in HCC screening programs for patients with advanced fibrosis (F3) or cirrhosis identified through biopsy or elastography, irrespective of etiology. In contrast, the American society proposes initiating screening upon confirmation of advanced fibrosis by two non-invasive methods. These methods are categorized into three groups: imaging techniques (e.g., VCTE or magnetic resonance imaging), point-of-care tests combining demographic and analytical parameters (e.g., FIB-4 or NAFLD fibrosis score), and specific blood tests (e.g., Enhanced Liver Fibrosis panel or Fibrospect 2). Additionally, the American guideline specifies that tests utilized should belong to different groups [35,36,37].

### 3.5. Prevention

Weight loss interventions have shown promise in mitigating the progression of MASLD, potentially reducing steatosis, steatohepatitis, and fibrosis. Evidence suggests that weight loss medications like orlistat and GLP-1 receptor agonists, such as liraglutide, as well as bariatric surgery, are associated with a decreased incidence of HCC [29]. Moreover, dietary interventions play a crucial role in HCC prevention, with the Mediterranean diet often recommended due to its rich fiber, unsaturated fats, and vitamin content. Higher adherence to this diet has been linked to a reduced risk of HCC in several studies. However, there are some discrepancies. For example, in an American prospective study, there was no significant association between the Mediterranean diet and the development of HCC (HR 0.75; 95% CI 0.49–1.15) [45]. Additionally, the Mediterranean diet offers added benefits in improving comorbidities associated with MASLD [29,45,46]. Coffee consumption has been proposed as a preventive lifestyle measure in MASLD. In a metanalysis of six Japanese cohort studies, regular coffee consumption was associated with a relative risk of hepatocellular carcinoma at 0.5 (95% CI, 0.38–0.66) [45].

Physical activity has emerged as another protective factor against HCC. In the EPIC study, regular physical activity was associated with a 45% reduction in HCC risk (RR 0.55, 95% CI 0.38–0.80), independent of other risk factors [8,47]. Furthermore, lifestyle modifications, such as the cessation of alcohol and tobacco consumption, are essential for HCC prevention in MASLD patients [29,45,48]. Alcohol cessation reduces HCC development risk by 6–7% a year [48].

Managing glycemic control is also of paramount importance in MASLD and metabolic syndrome. Oral antidiabetic medications, particularly metformin, have shown promise in reducing HCC risk, potentially through the reduction of IGF-1 levels. In a metanalysis of 24 studies, metformin was associated with a 41% lower risk of HCC in diabetic patients (*p* < 0.001) and lower mortality (Hr 0.74, 95% CI 0.66–0.82, *p* = 0.037) [29]. However, insulin or sulfonylureas may pose a higher risk [40,45,49]. Likewise, the treatment of dyslipidemia with statins appears to decrease the risk of HCC, particularly with lipophilic statins like atorvastatin and simvastatin. This risk reduction is believed to be dose-dependent and attributed to their anti-inflammatory, antiangiogenic, and antiproliferative properties [40,46,48,50]. The protective role of statins is reflected in multiple studies. In a metanalysis carried out by Islam et al. that included 24 studies, treatment with statins was associated with a decreased risk of developing hepatocellular carcinoma (RR 0.54, 95% CI: 0.30–0.42) [50]. On the other hand, regular aspirin use, commonly employed for cardiovascular prevention, may also reduce HCC risk, due to its anti-inflammatory effects and inhibition of COX-2. Studies have shown a significant reduction in HCC risk with regular aspirin use. For example, in a prospective study carried out in the USA, the consumption of at least 650 mg of aspirin per week was associated with a lower risk of developing HCC (HR 0.51; 95% CI 0.34–0.77) [45,46].

Considering the microbiome’s role in HCC pathogenesis, the use of pre- and probiotics for prevention in MASLD has been proposed [45].

These supplements potentially decrease inflammation, improve intestinal barrier integrity, and modulate immune response, with minimal adverse effects. However, further research is needed to identify the most beneficial species for this condition [45].

Patients with MASLD who develop fibrosis have a higher risk of developing HCC, so drugs that slow this progression could have a protective effect for HCC. Different antifibrotic drugs have been developed, such as obeticholic acid, which reduces fibrosis, but its role in the prevention of HCC is not yet known [45,48].

### 3.6. Treatment of HCC Related to MASLD

To date, the therapeutic options for HCC related to MASLD are the same as for other etiologies, including locoregional and systemic therapies [35,36]. However, the characteristics of this group of patients, both due to the usual presence of multiple cardiovascular risk factors and the diagnosis at older ages, may contraindicate some treatments. In some series, it is described that these patients have a higher use of liver resection, which may be related to a non-negligible proportion of HCC in the absence of underlying cirrhosis. Surgery is a curative treatment for single lesions. It is feasible in non-cirrhotic patients and in patients with cirrhosis without decompensation or significant portal hypertension. It must be taken into account that the frequent cardiovascular comorbidities in this group can increase the surgical risk, so the risk–benefit must be evaluated prior to treatment. Liver transplantation is a less used option in this group, mainly due to the larger tumor size at diagnosis, advanced age, and comorbidities. Moreover, the mortality on the waiting list is higher, mainly due to cardiovascular risk factors related to MASLD. Also, obesity, especially when it comes to morbid obesity (with a BMI greater than 40 kg/m^2^), may contraindicate transplantation [49]. Systemic therapies include multi-tyrosine kinase inhibitors (mTKIs), which inhibit tumor growth and have anti-angiogenic effects. Sorafenib was the first systemic treatment used in HCC. The SHARP study showed that compared to the placebo, it increased survival. However, due to its many adverse effects, sorafenib is not currently used. Lenvatinib presented non-inferiority results compared to sorafenib in the REFLECT study and is currently the first-line treatment in patients who are not candidates for curative treatment [35,36].

The available evidence suggests that immunotherapy treatments may exhibit reduced efficacy in patients with MASLD-HCC [51]. However, these findings may be influenced by biases, as most studies compare patients with HCC of viral etiology with those of other etiologies, including MASLD, as well as alcohol consumption and autoimmune causes. This discrepancy could stem from the immune alterations present in MASLD patients. Notably, the microbiota is thought to play a critical role, with some authors even proposing investigating fecal microbiota transplantation as a potential treatment for MASLD-HCC [51]. Despite these challenges, there is a consensus among researchers regarding the necessity of ongoing research to identify new therapeutic targets and to develop more tailored treatments [49,51].

## 4. Discussion

The etiology of liver diseases has changed over the last few decades. Previously, viral hepatitis predominated, but currently, we are facing an exponential growth of liver disease related to metabolic syndrome, in line with the global obesity pandemic. Its new nomenclature, MASLD, better reflects the relationship with this syndrome and its systemic consequences [4].

The current guidelines of the main scientific societies of hepatology are not specific for MASLD. These patients have some particularities compared to other etiologies, mainly older age, higher prevalence of cardiovascular pathology, and metabolic syndrome [35,36]. It is worth noting that according to some studies, up to 40% of the cases of HCC related to MASLD develop in patients who do not have liver cirrhosis [27]. In the analysis conducted by Hao Tan et al., in the cases of HCC in the group of patients with MASLD, only 33% were enrolled in screening programs, while in other pathologies, it reached 56% [28]. Understanding the barriers to screening in MASLD patients, such as healthcare access issues or physician awareness, would provide more comprehensive insights about these disparities. This significant disparity prompts the question: should all MASLD patients undergo HCC screening, even in the absence of cirrhosis? As of now, the answer is no. There is consensus that efforts should focus on developing tools to identify those at higher risk of developing HCC. Thus, current research focuses on the development and validation of various tests and indices to detect advanced fibrosis, as it is known that the risk of HCC parallels liver fibrosis. To date, FIB-4, NFS, and GALAD are laboratory-based scores that provide good specificity and sensitivity in predicting HCC [4,29,35,36,37]. However, they are currently considered complementary tools and are not incorporated into algorithms, due to the limited validation studies available. Critical analysis is needed to find out the sensitivity, specificity, and clinical utility of these tools in MASLD patients, compared to other etiologies.

The current guidelines from major hepatology societies worldwide recommend screening only in cases of advanced fibrosis, with minor variations between them and without differentiation based on etiology. As stated above, those guidelines, originally developed for viral hepatitis, which historically dominated liver disease worldwide, may need to adapt to the increasing prevalence of MASLD or include specific recommendations for this patient group, given its unique characteristics. In this regard, we propose that leading scientific societies should develop a consensus—possibly through Delphi studies or similar methodologies—to thoroughly assess and revise current guidelines. This process will ensure that any adaptations are grounded in robust scientific evidence and consensus, thereby enhancing the practical and clinical utility of these guidelines for managing MASLD.

Various pharmacological and non-pharmacological strategies have been proposed for preventing HCC related to MASLD. However, large-scale controlled prospective studies are necessary to establish general recommendations. While exercise stands out among non-pharmacological measures, specific guidelines are lacking. Regarding chemoprophylaxis, metformin, aspirin, and statins are prominent options, deemed safe drugs with the potential to reduce HCC incidence. Nevertheless, prospective controlled studies are required to assess their risk–benefit profile for this indication, considering that many MASLD patients already receive these medications due to their common comorbidities. Although numerous interventions are under investigation for HCC prevention in this population, it may be more cost-effective to prioritize primary prevention efforts for MASLD.

## 5. Conclusions

The prevalence of MASLD is on the rise, leading to an expected increase in hepatocellular carcinoma cases linked to MASLD. This condition more commonly affects older individuals who often have multiple comorbidities and typically present with larger lesions at diagnosis. Research is advancing on therapeutic strategies aimed at preventing HCC across various populations and demographics. However, up to 40% of HCC cases associated with MASLD occur in patients without liver cirrhosis, making them unsuitable for conventional ultrasound screening. Efforts are underway to develop non-invasive serological and imaging indices to identify high-risk individuals and to tailor HCC screening for those without cirrhosis. It is important to note that prospective, controlled studies are needed to provide robust evidence and validate screening strategies in MASLD-related HCC.

## 6. Future Directions

Developing risk stratification systems for HCC in MASLD patients without liver cirrhosis is essential. The validation of non-invasive risk prediction tools and the development of specific screening algorithms for MASLD are also equally crucial. Additionally, further research is needed to understand the etiopathogenesis of HCC in MASLD to identify targeted therapeutic options.

## References

[1] Ludwig J., Viggiano T.R., McGill D.B., Oh B.J. (1980). Nonalcoholic steatohepatitis: Mayo Clinic experiences with a hitherto unnamed disease. Mayo Clin. Proc..

[2] Han S.K., Baik S.K., Kim M.Y. (2023). Non-alcoholic fatty liver disease: Definition and subtypes. Clin. Mol. Hepatol..

[3] Eslam M., Newsome P.N., Sarin S.K., Anstee Q.M., Targher G., Romero-Gomez M., Zelber-Sagi S., Wong V.W.-S., Dufour J.-F., Schattenberg J.M. (2020). A new definition for metabolic dysfunction-associated fatty liver disease: An international expert consensus statement. J. Hepatol..

[4] Rinella M.E., Lazarus J.V., Ratziu V., Francque S.M., Sanyal A.J., Kanwal F., Romero D., Abdelmalek M.F., Anstee Q.M., Arab J.P. (2023). A multisociety Delphi consensus statement on new fatty liver disease nomenclature. Hepatology.

[5] Younossi Z.M., Paik J.M., Stepanova M., Ong J., Alqahtani S., Henry L. (2024). Clinical profiles and mortality rates are similar for metabolic dysfunction-associated steatotic liver disease and nonalcoholic fatty liver disease. J. Hepatol..

[6] Song S.J., Lai J.C., Wong G.L., Wong V.W., Yip T.C. (2023). Can we use old NAFLD data under the new MASLD definition?. J. Hepatol..

[7] Tinajero M.G., Malik V.S. (2021). An update on the epidemiology of type 2 diabetes: A global perspective. Endocrinol. Metab. Clin. N. Am..

[8] Younossi Z.M., Golabi P., Paik J.M., Henry A., Van Dongen C., Henry L. (2023). The global epidemiology of nonalcoholic fatty liver disease (NAFLD) and nonalcoholic steatohepatitis (NASH): A systematic review. Hepatology.

[9] Kim D., Touros A., Kim W.R. (2018). Nonalcoholic Fatty Liver Disease and Metabolic Syndrome. Clin. Liver Dis..

[10] Qureshi K., Abrams G.A. (2007). Metabolic liver disease of obesity and role of adipose tissue in the pathogenesis of nonalcoholic fatty liver disease. World, J. Gastroenterol..

[11] Buzzetti E., Pinzani M., Tsochatzis E.A. (2016). The multiple-hit pathogenesis of non-alcoholic fatty liver disease (NAFLD). Metabolism.

[12] Wasilewska N., Lebensztejn D.M. (2021). Non-alcoholic fatty liver disease and lipotoxicity. Clin. Exp. Hepatol..

[13] Cannito S., Morello E., Bocca C., Foglia B., Benetti E., Novo E., Chiazza F., Rogazzo M., Fantozzi R., Povero D. (2017). Microvesicules released from fat-laden cells promote activation of hepatocellular NLRP3 inflammasome: A pro-inflammatory link between lipotoxicity and non-alcoholic steatohepatitis. PLoS ONE.

[14] Miao X., Alidadipour A., Saed V., Sayyadi F., Jadidi Y., Davoudi M., Amraee F., Jadidi N., Afrisham R. (2024). Hepatokines: Unveiling the molecular and cellular mechanisms connecting hepatic tissue to insulin resistance and inflammation. Acta Diabetol..

[15] Zhou X.-D., Targher G., Byrne C.D., Somers V., Kim S.U., Chahal C.A.A., Wong V.W.-S., Cai J., Shapiro M.D., Eslam M. (2023). An international multidisciplinary consensus statement on MAFLD and the risk of CVD. Hepatol. Int..

[16] Yoneda M., Yamamoto T., Honda Y., Imajo K., Ogawa Y., Kessoku T., Kobayashi T., Nogami A., Higurashi T., Kato S. (2021). Risk of cardiovascular disease in patients with fatty liver disease as defined from the metabolic dysfunction associated fatty liver disease or nonalcoholic fatty liver disease point of view: A retrospective nationwide claims database study in Japan. J. Gastroenterol..

[17] Zhou X.-D., Cai J., Targher G., Byrne C.D., Shapiro M.D., Sung K.-C., Somers V.K., Chahal C.A.A., George J., Chen L.-L. (2022). Metabolic dysfunction-associated fatty liver disease and implications for cardiovascular risk and disease prevention. Cardiovasc. Diabetol..

[18] Gaiani K., Jornayvaz F.R. (2021). Pathophysiology of NASH in endocrine diseases. Endocr. Connect..

[19] Sanna C., Rosso C., Marietti M., Bugianesi E. (2016). Non-Alcoholic Fatty Liver Disease and Extra-Hepatic Cancers. Int. J. Mol. Sci..

[20] Mitsala A., Tsalikidis C., Romanidis K., Pitiakoudis M. (2022). Non-Alcoholic Fatty Liver Disease and Extrahepatic Cancers: A Wolf in Sheep’s Clothing?. Curr. Oncol..

[21] Streba L.A.M., Vere C.C., Rogoveanu I., Streba C.T. (2015). Nonalcoholic fatty liver disease, metabolic risk factors, and hepatocellular carcinoma: An open question. World J. Gastroenterol..

[22] Vetrano E., Rinaldi L., Mormone A., Giorgione C., Galiero R., Caturano A., Nevola R., Marfella R., Sasso F.C. (2023). Non-alcoholic fatty liver disease (NAFLD), Type 2 diabetes and non-viral hepatocarcinoma: Pathophysiological mechanisms and new therapeutic strategies. Biomedicines.

[23] Takakura K., Oikawa T., Nakano M., Saeki C., Torisu Y., Kajihara M., Saruta M. (2019). Recent insights into the multiple pathways driving non-alcoholic steatohepatitis-derived hepatocellular carcinoma. Front. Oncol..

[24] Huang D.Q., El-Serag H.B., Loomba R. (2020). Global epidemiology of NAFLD-related HCC: Trends, predictions, risk factors and prevention. Nat. Rev. Gastroenterol. Hepatol..

[25] Behary J., Amorim N., Jiang X.-T., Raposo A., Gong L., McGovern E., Ibrahim R., Chu F., Stephens C., Jebeili H. (2021). Gut microbiota impact on the peripheral immune response in non-alcoholic fatty liver disease related hepatocellular carcinoma. Nat. Commun..

[26] Kanda T., Goto T., Hirotsu Y., Masuzaki R., Moriyama M., Omata M. (2020). Molecular mechanisms: Connections between nonal-coholic fatty liver disease, steatohepatitis and hepatocellular carcinoma. Int. J. Mol. Sci..

[27] McGlynn K.A., Petrick J.L., El-Serag H.B. (2021). Epidemiology of Hepatocellular Carcinoma. Hepatology.

[28] Tan D.J.H., Ng C.H., Lin S.Y., Pan X.H., Tay P., Lim W.H., Teng M., Syn N., Lim G., Yong J.N. (2022). Clinical characteristics, surveillance, treatment allocation, and outcomes of non-alcoholic fatty liver disease-related hepatocellular carcinoma: A systematic review and meta-analysis. Lancet Oncol..

[29] Cernea S., Onișor D. (2023). Screening and interventions to prevent nonalcoholic fatty liver disease/nonalcoholic steatohepatitis-associated hepatocellular carcinoma. World J. Gastroenterol..

[30] Jarvis H., Craig D., Barker R., Spiers G., Stow D., Anstee Q.M., Hanratty B. (2020). Metabolic risk factors and incident advanced liver disease in non-alcoholic fatty liver disease (NAFLD): A systematic review and meta-analysis of population-based observational studies. PLoS Med..

[31] Chen Y.-T., Chen T.-I., Yang T.-H., Yin S.-C., Lu S.-N., Liu X.-R., Gao Y.-Z., Lin C.-J., Huang C.-W., Huang J.-F. (2024). Long-term Risks of Cirrhosis and Hepatocellular Carcinoma Across Steatotic Liver Disease Subtypes. Am. J. Gastroenterol..

[32] Yasui K., Hashimoto E., Komorizono Y., Koike K., Arii S., Imai Y., Shima T., Kanbara Y., Saibara T., Mori T. (2011). Characteristics of Patients with Nonalcoholic Steatohepatitis Who Develop Hepatocellular Carcinoma. Clin. Gastroenterol. Hepatol..

[33] Vitale A., Svegliati-Baroni G., Ortolani A., Cucco M., Dalla Riva G.V., Giannini E.G., Piscaglia F., Rapaccini G., Di Marco M., Caturelli E. (2023). Epidemiological trends and trajectories of MAFLD-associated hepatocellular carcinoma 2002–2033: The ITA.LI.CA database. Gut.

[34] Stine J.G., Wentworth B.J., Zimmet A., Rinella M.E., Loomba R., Caldwell S.H., Argo C.K. (2018). Systematic review with meta-analysis: Risk of hepatocellular carcinoma in non-alcoholic steatohepatitis without cirrhosis compared to other liver diseases. Aliment. Pharmacol. Ther..

[35] Loomba R., Lim J.K., Patton H., El-Serag H.B. (2020). AGA Clinical Practice Update on Screening and Surveillance for Hepatocellular Carcinoma in Patients with Nonalcoholic Fatty Liver Disease: Expert Review. Gastroenterology.

[36] (2018). EASL clinical practice guidelines: Management of hepatocellular carcinoma. J. Hepatol..

[37] Taru M.-G., Lupsor-Platon M. (2023). Exploring Opportunities to Enhance the Screening and Surveillance of Hepatocellular Carcinoma in Non-Alcoholic Fatty Liver Disease (NAFLD) through Risk Stratification Algorithms Incorporating Ultrasound Elastography. Cancers.

[38] Machado M.V. (2023). The Growing Landscape of NAFLD-Associated Hepatocellular Carcinoma and Its Impact in Surveillance. GE-Port. J. Gastroenterol..

[39] Alexander M., Loomis A.K., Van Der Lei J., Duarte-Salles T., Prieto-Alhambra D., Ansell D., Pasqua A., Lapi F., Rijnbeek P., Mosseveld M. (2019). Risks and clinical predic-tors of cirrhosis and hepatocellular carcinoma diagnoses in adults with diagnosed NAFLD: Real-world study of 18 million pa-tients in four European cohorts. BMC Med..

[40] Shah P.A., Patil R., Harrison S.A. (2023). NAFLD-related hepatocellular carcinoma: The growing challenge. Hepatology.

[41] Cagnin S., Donghia R., Martini A., Pesole P.L., Coletta S., Shahini E., Boninsegna G., Biasiolo A., Pontisso P., Giannelli G. (2023). Galad Score as a Prognostic Marker for Patients with Hepatocellular Carcinoma. Int. J. Mol. Sci..

[42] Guan M.-C., Zhang S.-Y., Ding Q., Li N., Fu T.-T., Zhang G.-X., He Q.-Q., Shen F., Yang T., Zhu H. (2023). The Performance of GALAD Score for Diagnosing Hepatocellular Carcinoma in Patients with Chronic Liver Diseases: A Systematic Review and Meta-Analysis. J. Clin. Med..

[43] Berhane S., Toyoda H., Tada T., Kumada T., Kagebayashi C., Satomura S., Schweitzer N., Vogel A., Manns M.P., Benckert J. (2016). Role of the GALAD and BALAD-2 Serologic Models in Diagnosis of Hepatocellular Carcinoma and Prediction of Survival in Patients. Clin. Gastroenterol. Hepatol..

[44] Chen V.L., Xu D., Wicha M.S., Lok A.S., Parikh N.D. (2020). Uility of liquid biopsy analysis in detection of hepatocellular carcinoma, determination of prognosis, and disease monitoring: A systematic review. Clin. Gastroenterol. Hepatol..

[45] Lange N.F., Radu P., Dufour J.-F. (2021). Prevention of NAFLD-associated HCC: Role of lifestyle and chemoprevention. J. Hepatol..

[46] Geh D., Anstee Q.M., Reeves H.L. (2021). NAFLD-Associated HCC: Progress and Opportunities. J. Hepatocell. Carcinoma.

[47] Baumeister S.E., Schlesinger S., Aleksandrova K., Jochem C., Jenab M., Gunter M.J., Overvad K., Tjønneland A., Boutron-Ruault M.C., Carbonnel F. (2019). Association between physical activity and risk of hepatobiliary cancers: A multinational cohort study. J. Hepatol..

[48] Ahmad M.I., Khan M.U., Kodali S., Shetty A., Bell S.M., Victor D. (2022). Hepatocellular Carcinoma Due to Nonalcoholic Fatty Liver Disease: Current Concepts and Future Challenges. J. Hepatocell. Carcinoma.

[49] Marengo A., Rosso C., Bugianesi E. (2016). Liver Cancer: Connections with Obesity, Fatty Liver, and Cirrhosis. Annu. Rev. Med..

[50] Islam M.M., Poly T.N., Walther B.A., Yang H.-C., Li Y.-C.J. (2020). Statin Use and the Risk of Hepatocellular Carcinoma: A Meta-Analysis of Observational Studies. Cancers.

[51] Pinter M., Pinato D.J., Ramadori P., Heikenwalder M. (2023). NASH and Hepatocellular carcinoma: Immunology and immunotheray. Clin. Cancer Res..

